# Screening prognostic markers for hepatocellular carcinoma based on pyroptosis-related lncRNA pairs

**DOI:** 10.1186/s12859-023-05299-9

**Published:** 2023-04-29

**Authors:** Tong Wu, Na Li, Fengyuan Luo, Zhihong Chen, Liyuan Ma, Tao Hu, Guini Hong, Hongdong Li

**Affiliations:** 1 School of Medical Information Engineering, Gannan Medical University, Ganzhou, 341000 China; 2 School of Public Health and Health Management, Gannan Medical University, Ganzhou, 341000 China

**Keywords:** Hepatocellular carcinoma, Pyroptosis, Long noncoding RNA, Prognosis, Relative expression ordering

## Abstract

**Background:**

Pyroptosis is closely related to cancer prognosis. In this study, we tried to construct an individualized prognostic risk model for hepatocellular carcinoma (HCC) based on within-sample relative expression orderings (REOs) of pyroptosis-related lncRNAs (PRlncRNAs).

**Methods:**

RNA-seq data of 343 HCC samples derived from The Cancer Genome Atlas (TCGA) database were analyzed. PRlncRNAs were detected based on differentially expressed lncRNAs between sample groups clustered by 40 reported pyroptosis-related genes (PRGs). Univariate Cox regression was used to screen out prognosis-related PRlncRNA pairs. Then, based on REOs of prognosis-related PRlncRNA pairs, a risk model for HCC was constructed by combining LASSO and stepwise multivariate Cox regression analysis. Finally, a prognosis-related competing endogenous RNA (ceRNA) network was built based on information about lncRNA–miRNA–mRNA interactions derived from the miRNet and TargetScan databases.

**Results:**

Hierarchical clustering of HCC patients according to the 40 PRGs identified two groups with a significant survival difference (Kaplan–Meier log-rank, *p* = 0.026). Between the two groups, 104 differentially expressed lncRNAs were identified (|log_2_(FC)|> 1 and FDR < 5%). Among them, 83 PRlncRNA pairs showed significant associations between their REOs within HCC samples and overall survival (Univariate Cox regression, *p* < 0.005). An optimal 11-PRlncRNA-pair prognostic risk model was constructed for HCC. The areas under the curves (AUCs) of time-dependent receiver operating characteristic (ROC) curves of the risk model for 1-, 3-, and 5-year survival were 0.737, 0.705, and 0.797 in the validation set, respectively. Gene Set Enrichment Analysis showed that inflammation-related interleukin signaling pathways were upregulated in the predicted high-risk group (*p* < 0.05). Tumor immune infiltration analysis revealed a higher abundance of regulatory T cells (Tregs) and M2 macrophages and a lower abundance of CD8 + T cells in the high-risk group, indicating that excessive pyroptosis might occur in high-risk patients. Finally, eleven lncRNA–miRNA–mRNA regulatory axes associated with pyroptosis were established.

**Conclusion:**

Our risk model allowed us to determine the robustness of the REO-based PRlncRNA prognostic biomarkers in the stratification of HCC patients at high and low risk. The model is also helpful for understanding the molecular mechanisms between pyroptosis and HCC prognosis. High-risk patients may have excessive pyroptosis and thus be less sensitive to immune therapy.

**Supplementary Information:**

The online version contains supplementary material available at 10.1186/s12859-023-05299-9.

## Background

Hepatocellular carcinoma (HCC) is one of the most common human malignancies, with over 800,000 new cases and nearly 700,000 deaths worldwide yearly [1]. HCC is highly heterogeneous and insidious; most patients are diagnosed at advanced stages with poor prognosis [2]. According to statistics, the overall median survival time of patients with advanced HCC is only 9 months, and the 5-year overall survival (OS) is solely 10% [3]. Therefore, exploring the molecular biomarkers associated with HCC prognosis has been a hot issue in HCC research [4].

Pyroptosis is a sort of programmed cell death related to inflammation, which is mediated by the intracellular inflammasome and gasdermins [5, 6]. Appropriate induction of pyroptosis could trigger a moderate inflammatory reaction that might enhance innate immunity and generate an antitumor immune response [7, 8]. In contrast, excessive pyroptosis might excite a hyperinflammatory response that disrupts immune homeostasis and promotes cancer progression [7, 8]. Consequently, some researchers attempt to improve the prognosis of tumor patients by regulating the activation of pyroptosis to produce antitumor immune responses [9]. For instance, migration and invasion of oral squamous cell carcinoma cells could be inhibited via pyroptosis activation by anthocyanidins [10]. High expression of gasdermin E induced by miltirone could be used to provoke pyroptosis in cancer cells [11]. NLRP3 (NLR Family Pyrin Domain Containing 3) inflammasomes could be utilized to mediate pyroptosis to suppress the growth and metastasis of HCC cells [12]. These suggest pyroptosis is closely associated with cancer prognosis.

Long noncoding RNA (lncRNA) is a transcription product of DNA with a length greater than 200 nucleotides, which can regulate gene expression by interacting with proteins, DNA, or other RNAs [13]. It has been reported that lncRNAs are critical regulators of pyroptosis [14]. For example, lncRNA HOTTIP could inhibit pyroptosis and promote ovarian cancer cell proliferation by targeting miR-148a-3p/AKT2 axis [15]. LncRNA MEG3 could inhibit the growth and metastasis of triple-negative breast cancer by activating pyroptosis via NLRP3/caspase-1/GSDMD pathway [16]. These studies demonstrate that aberrant alterations of pyroptosis-related lncRNAs (PRlncRNAs) also impact cancer prognosis.

Prognosis-associated PRlncRNA biomarkers have been identified for multiple cancer types. For HCC, seven and five prognosis-associated PRlncRNAs have been reported [17, 18]. These PRlncRNA prognostic risk models have some efficacy in training and testing datasets. However, their risk thresholds are summarized from the absolute expression levels of PRlncRNAs, which are often data-dependent and unstable, leading to difficulties when applied in clinical settings [19]. In recent years, researchers have found that the within-sample relative expression orderings (REOs) of genes were more robust than the absolute expression levels of genes across samples. Furthermore, the REO-based molecular biomarkers can be easily applied to individual diagnosis, which is more suitable for clinics [20–22].

In this study, we tried identifying REO-based prognosis-associated PRlncRNA biomarkers to construct an individualized prognostic risk model for HCC and explore the molecular mechanisms between pyroptosis and HCC prognosis.

## Materials and methods

### Data collection and preprocessing

The RNA expression data of the 371 HCC and 50 adjacent normal tissue samples analyzed in this study were downloaded from The Cancer Genome Atlas (TCGA) database. The data were obtained using Illumina HiSeq 2000 RNA Sequencing technology. A total of 60,483 RNAs were detected. The data were preprocessed by the following procedures. Firstly, remove the samples with a survival time of less than 30 days or missing survival time. Secondly, normalize expression values to Transcript Per Million (TPM). Thirdly, annotate each examined RNA in the GENCODE database. Finally, exclude mRNAs with count values less than 1 and lncRNAs with count values less than 0.5 in all samples.

For miRNA data downloaded from the TCGA database, sequencing was performed on an Illumina HiSeq miRNASeq platform. After deleting the miRNAs with a count value of 0 in more than 50% of the samples, 578 miRNAs were kept. The miRNA expression values were normalized to Reads Per Million (RPM).

### Detection of prognosis-related lncRNA pairs

Clustering HCC samples with the ward linkage algorithm were performed on 40 pyroptosis-related genes (PRGs), which were derived from the pyroptosis pathway in the Molecular Signatures Database (MSigDB) (Additional file 1: Table S1). Then, differentially expressed lncRNAs detected between the two HCC groups were defined as pyroptosis-related lncRNAs. Any two pyroptosis-related lncRNAs can form a lncRNA pair. For a lncRNA pair (lncRNA_i_|lncRNA_j_), there are two REO status, lncRNA_i_ < lncRNA_j_ or lncRNA_i_ ≥ lncRNA_j_, in a sample. The prognosis-related lncRNA pairs were then identified by the following procedures.

1. Combine the lncRNAs of interest two by two to form ${C}_{k}^{2}$ (*k* is the number of pyroptosis-related lncRNAs) lncRNA pairs.
2. The REO matrix, *X*, was constructed based on lncRNA pairs for the training set. *X*_*ij*_ denoted the REO of the *i*-th lncRNA pair (lncRNA_*i*1_|lncRNA_*i*2_) in the *j*-th sample, taking 1 or 0, with 1 representing lncRNA_*i*1_ < lncRNA_*i*2_ and 0 representing lncRNA_*i*1_ ≥ lncRNA_*i*2_.
3. Remove lncRNA pairs for which the percentage of REOs with 1 was less than 20% or greater than 80%. This criterion ensured that the apparent reversal of REOs of lncRNA pairs occurred in a certain amount of HCC samples to facilitate the identification of sample subgroups with different prognoses, as lncRNA pairs with the same score (0 or 1) in more than 80% of samples were considered uninformative [21].
4. For each remaining lncRNA pair, the correlation between its REO values and OS times was evaluated by univariate Cox regression. If the Wald test's *p* value is less than 0.005, the lncRNA pair was considered as a prognosis-related lncRNA pair.

### Construction and evaluation of prognostic lncRNA pair risk model

The LASSO regression and stepwise multivariate Cox regression algorithms were applied to select candidate PRlncRNA pairs as prognostic biomarkers. LASSO regression was adopted to choose the prognosis-related PRlncRNA pairs most predictive of OS. Lambda values corresponding to the smallest partial likelihood deviance were chosen as the optimal parameters after tenfold cross-validation [23, 24]. Then, multivariate Cox regression analysis based on the Akaike information criterion (AIC) method was used to determine the optimal model. The model with the lowest AIC value was considered as the optimal prognostic risk model, with the corresponding PRlncRNA pairs as the eventual predictive risk biomarkers [25].

The time-dependent receiver operating characteristic (ROC) curves were applied to assess the performance, and the Youden index determined the risk threshold. Multivariate Cox proportional hazards regression analysis was employed to evaluate independent prognostic factors associated with OS [26]. Covariates included risk scores for prognostic PRlncRNA pairs, gender, age, tumor stage, and grade.

### Enrichment analysis and estimation of immune cell infiltration

Functional enrichment analysis of differentially expressed genes between the high- and low-risk groups was completed by Gene Set Enrichment Analysis (GSEA) based on the Reactome database with annotation information from the MSigDB database (v7.5.1).

The estimation of the absolute abundance of tumor-infiltrating (immune cells in HCC samples was achieved by the CIBERSORT algorithm [27].

### Construction of prognostic pyroptosis-related competing endogenous RNA (ceRNA) network

The regulatory relationships of lncRNAs, miRNAs, and mRNAs were obtained from the miRNet database and the TargetScan database [28, 29]. The miRNet database was utilized to predict target miRNAs for lncRNAs, and the TargetScan database was used to predict target miRNAs for PRGs.

### Statistical analysis

All statistical analysis were completed with R 4.1.0 software. Cluster analysis was finished with the ward linkage algorithm. Differential expression analysis was implemented with the "*limma*"package. Survival analysis and corresponding plotting were based on the "*survival*", "*glmnet*", "*MASS*" and "*survminer*" packages. ROC analysis and the determination of risk threshold were completed based on the "*survivalROC*" package. GSEA was based on the "*clusterProfiler*" package. Tumor immune infiltration analysis was implemented by the "*immunedeconv*" package. The Benjamini–Hochberg (BH) method was applied to control the false discovery rate (FDR). Unless otherwise specified, the statistical significance level was set uniformly at 0.05.

## Results

### Pyroptosis-related lncRNAs

**Fig. 1:**
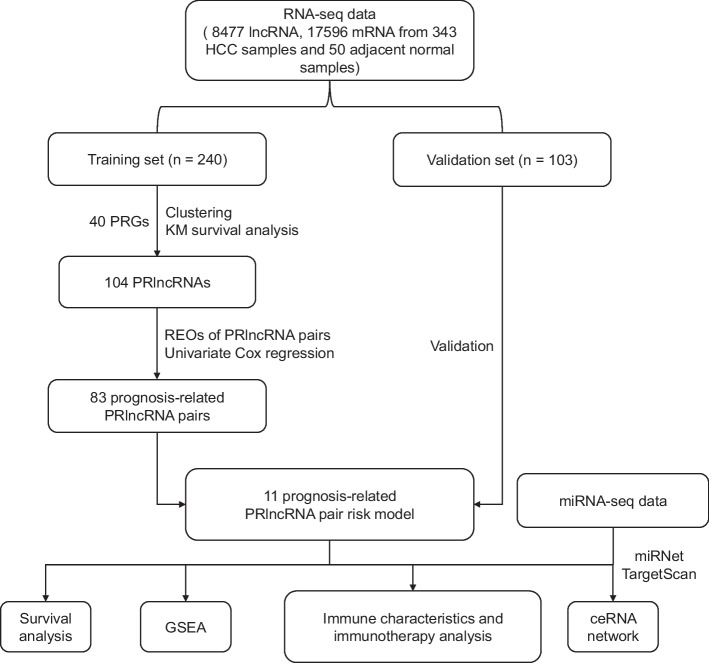
Workflow of this study

The workflow of this study is illustrated in Fig. 1. After data preprocessing, the TCGA HCC data set included expression measurements of 8477 lncRNAs and 17,596 mRNAs from 343 HCC samples. Firstly, all samples were randomly categorized into training (*n* = 240) and validation (*n* = 103) sets. No significant differences have been observed in the clinical features between the training and validation sets (*p* value < 0.05, Additional file 1: Table S2).

**Fig. 2:**
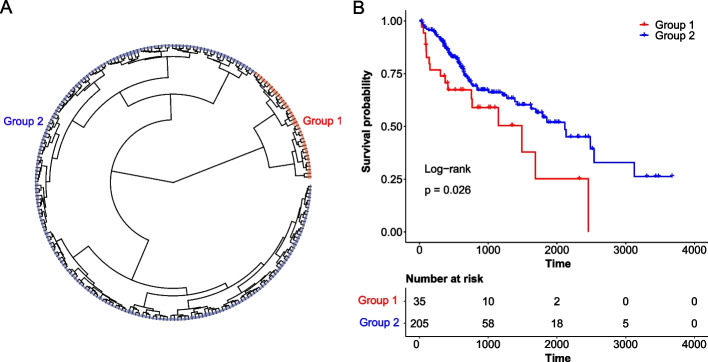
Hierarchical clustering and Kaplan–Meier survival analysis. **A** Hierarchical clustering for the 40 PRGs in the training set. **B** Kaplan–Meier survival curves for clusters derived from hierarchical clustering

Hierarchical clustering was performed on the expression levels of the 40 PRGs in the training set. Samples were clustered into two groups, containing 35 and 205 samples, respectively (Fig. 2A). Kaplan–Meier survival analysis revealed a significant difference in survival between these two groups of patients (log-rank test, *p* value = 0.026 (Fig. 2B)), which suggested that the expression pattern of PRGs was associated with the prognosis of HCC patients.

Using the R package "*limma*", 104 lncRNAs that were differentially expressed between the two groups were identified at |log_2_(FC)|> 1 and FDR < 5%. These 104 lncRNAs were considered as PRlncRNAs for the following analysis.

### Prognosis-related lncRNA pairs and risk model

**Fig. 3:**
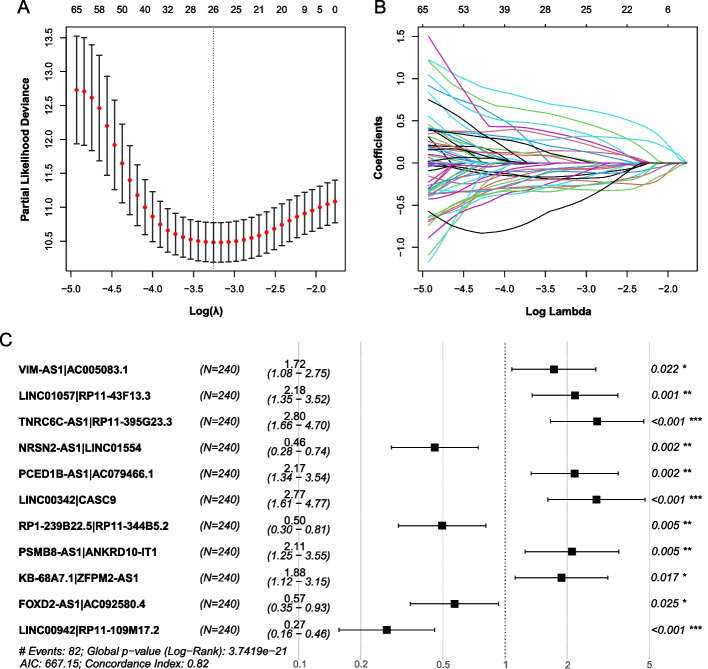
Establishment of the prognostic lncRNA pair risk model. **A** The plot of partial likelihood deviance versus log(λ). A vertical dotted line marks the smallest partial likelihood deviance; **B** LASSO coefficient profiles of HCC OS-associated lncRNA pairs; **C** forest plot depicting associations between lncRNA pairs and risk values determined by multivariate Cox regression analysis. *p < 0.05; **p < 0.01; ***p < 0.001

The 104 PRlncRNAs were paired, and prognosis-related lncRNA pairs were identified based on the within-sample REOs in the training set (see Methods). The REOs of 83 PRlncRNA pairs were observed to be significantly associated with the OS time of HCC by univariate Cox regression analysis. To choose representative prognosis-related lncRNA pairs, we performed the LASSO Cox regression analysis via tenfold cross-validation on these 83 PRlncRNA pairs, and 26 PRlncRNA pairs were selected at the smallest partial likelihood deviance (Fig. 3A, B). Then, the stepwise multivariate Cox regression analysis was performed on 26 PRlncRNA pairs to choose prognosis-related lncRNA pair biomarkers and construct the risk model. Finally, as shown in Fig. 3C, 11 PRlncRNA pairs involving 22 PRlncRNAs were selected at the smallest AIC value. The corresponding risk model is: risk score = 0.5447 × VIM-AS1|AC005083.1 + 0.7797 × LINC01057|RP11-43F13.3 + 1.0279 × TNRC6C-AS1|RP11-395G23.3 − 0.7839 × NRSN2-AS1|LINC01554 + 0.7766 × PCED1B-AS1|AC079466.1 + 1.0191 × LINC00342|CASC9 − 0.7017 × RP1-239B22.5|RP11-344B5.2 + 0.7464 × PSMB8-AS1|ANKRD10-IT1 + 0.6299 × KB-68A7.1|ZFPM2-AS1 − 0.5646 × FOXD2-AS1|AC092580.4 − 1.3199 × LINC00942|RP11-109M17.2 (Fig. 3C). The threshold for high- and low-risk groups was determined by the point with the largest Youden index on the 5-year ROC curve of the training set (Youden index = 0.739, risk score = 0.025), with 105 and 135 patients classified as high- and low-risk samples, respectively. Among the 22 PRlncRNAs, 16 were differentially expressed between high- and low-risk patients. Comparing all HCC samples to adjacent normal samples, 14 of these 16 PRlncRNAs were differentially expressed.

### Validation of prognostic lncRNA pair risk model

**Fig. 4:**
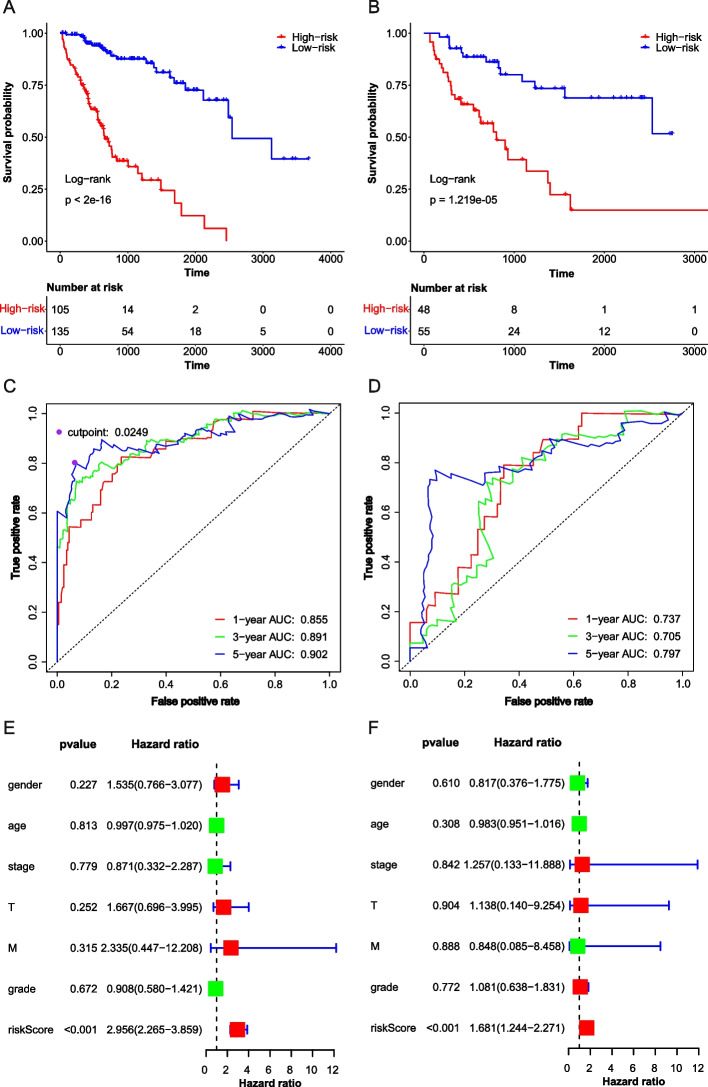
Validation of the prognostic lncRNA pair risk model. **A**, **B** Kaplan–Meier survival curves for high- and low-risk groups in the training and validation sets; **C**, **D** ROC curves based on the predictive efficacy of the lncRNA pair risk model for the training and validation sets; **E**, **F** Multivariate Cox regression analysis showed the independence of the risk model for the prediction for the training and validation sets

According to the risk threshold, the patients in the validation set were divided into a high-risk group (risk score ≥ 0.025) and a low-risk group (risk score < 0.025), respectively. Kaplan–Meier survival analysis showed that OS rates were significantly different between the high- and low-risk groups in both the training and validation sets (log-rank test: *p* value < 2 × 10^–16^ and *p* value = 1.219 × 10^–5^, Fig. 4A, B). The AUCs of time-dependent ROC curves showed the prediction accuracies of 1-, 3- and 5-year survival were 0.855, 0.891, and 0.902 for the training set, and 0.737, 0.705, and 0.797 for the validation set, respectively (Fig. 4C, D). In addition, the results of multivariate Cox regression indicated that the risk model was an independent prognostic factor for patients with HCC (*p* value < 0.001, Fig. 4E, F). These results suggest that the risk score model based on lncRNA pairs can be an efficient tool for predicting the prognostic risk of HCC.

### Analysis of immune-related characteristics of high- and low-risk groups

**Fig. 5:**
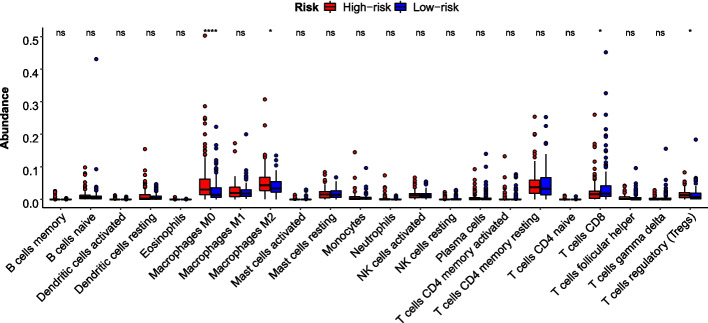
Immune infiltration analysis between high- and low-risk groups

Among the 40 PRGs, 27 were differentially expressed between high- and low-risk groups in the training set at FDR < 5%. Notably, 25 PRGs were significantly upregulated in the high-risk group. GSEA showed the inflammation-related interleukin-mediated signaling pathways, including the interleukin 4 and interleukin 13 signaling pathways (q-value = 0.021), and signaling by interleukins pathway (q-value = 0.030), were also found to be significantly upregulated in the high-risk group [8]. Furthermore, as shown in Fig. 5, the abundance of regulatory T cells (Tregs) and M2 macrophages was significantly higher in the high-risk group. In comparison, the abundance of CD8 + T cells was significantly lower in the high-risk group. These results suggested that the immune system might have overreacted in the high-risk group due to the upregulation of PRGs expression, disrupting the immune homeostasis and making the prognosis worse.

### Establishment of prognostic pyroptosis-related ceRNA network

**Fig. 6:**
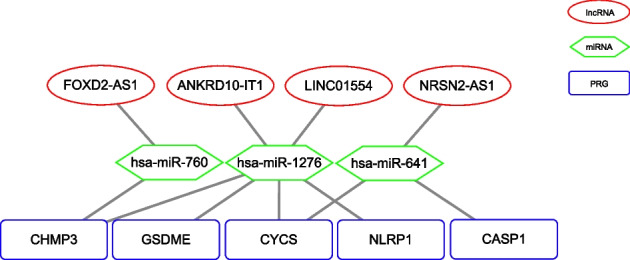
Pyroptosis-associated prognostic ceRNA network

The target miRNAs of the 22 lncRNAs involved in the 11 PRlncRNA pair biomarkers in the risk score model were predicted by the miRNet database. Finally, 140 target miRNAs of 10 lncRNAs were obtained. The TargetScan database showed that 112 of the 140 miRNAs targeted 39 PRGs. By univariate Cox regression, three of the 112 target miRNAs and eight of the 39 target PRGs were significantly associated with the prognosis of HCC in our data. Moreover, five of the eight PRGs were targets of the three miRNAs. These prognosis-related lncRNAs, miRNAs, and PRGs formed eleven lncRNA–miRNA–mRNA regulatory axes (Fig. 6), involving four lncRNAs, three miRNAs, and five PRGs.

## Discussion

In this study, we have constructed a prognostic risk model for HCC by survival analysis of PRlncRNA pairs based on the within-sample REOs of PRlncRNAs. The risk model has good predictive performance at classifying the HCC patients into high- and low-risk groups in the validation set. Given the use of within-sample REOs of PRlncRNA pairs, our prognostic risk model has potential implications for clinical translation and application. Our model is independent of systematic bias and suitable for the individualized clinic and may help to stratify HCC patients at high risk of poor prognosis.

The 240 training samples were clustered by 40 PRGs into two groups of samples with significantly different prognoses, containing 35 cases with relatively poor survival and 205 cases with relatively good survival, respectively. Moreover, these 240 samples were divided by our prognostic prediction model into 105 high-risk cases and 135 low-risk cases. They were significantly overlapped (*p* = 3.45 × 10^–4^, hypergeometric test): the 35 poor prognosis samples overlapped with the 105 high-risk samples by 25 and the 205 good prognosis samples overlapped with the 135 low-risk samples by 125. The discrepancy may be due to the different purposes of stratifying samples. The clustering of samples using PRGs was intended to identify differential lncRNAs as PRlncRNAs. The two groups clustered by them may reflect more the similarity of expression patterns of the samples across all PRGs. The high- and low-risk groups were stratified by the constructed prognostic model. They should be more associated with the prognosis of HCC.

We found that more PRGs were upregulated in the high-risk group compared with the low-risk group, indicating more inflammation responses in the high-risk group. Further evidence was provided by the pathway enrichment and immune infiltration analysis results. GSEA showed that interleukin-mediated inflammation-related signaling pathways were upregulated in the high-risk group. CIBERSORT-based analysis showed higher abundance of Tregs and M2 macrophages and a lower abundance of CD8 + T cells in the high-risk group. It has been reported that interleukin 4 and interleukin 13 signaling could induce type 2 inflammatory processes [30]. If the type 2 inflammatory responses were out of control, M2 polarization of macrophages could be promoted, effectively suppressing the cytotoxicity of CD8 + T cells and NK cells [30, 31]. Except for M2 macrophages, Tregs, which function could be enhanced by chronic inflammation, could also efficiently inhibit the function of CD8 + T cells [8, 32]. Relative low abundance of CD8 + T cells has been reported to indirectly induce weaker cytotoxicity, while lower cytotoxicity might induce more insensitivity to immunotherapy [33]. Therefore, we additionally analyzed the cytotoxic-related genes (GZMA, GZMB, GZMK, PRF1) [34] and observed downregulation of these genes in the high-risk group compared to low-risk group (Wilcoxon rank-sum test: *p* value < 0.05; *p* value < 0.05; *p* value < 0.05; *p* value < 0.001). We then applied the Immune Cell Abundance Identifier (ImmuCellAI) database to predict the immunotherapeutic responses. We found that patients in the high-risk group were likely to have lower scores and be less sensitive to immune checkpoint blockade therapy (Wilcoxon rank-sum test: *p* value = 0.010) [35]. Therefore, we inferred that excessive pyroptosis might have arisen in high-risk patients, reducing the amount and activity of tumor-infiltrating lymphocytes and worsening tumor prognosis [36].

In clinical practice, due to the simplicity and non-invasiveness, serum markers such as AFP, DCP, and AFP-L3 are often used to diagnose HCC and predict prognosis. Many scoring models have used the three markers for the diagnosis or prognosis of HCC. The GALAD, consisting of age, sex, and the three markers, has been reported to have high predictive accuracy for early HCC in patients with nonalcoholic steatohepatitis (AUC = 0.96) [37] and also accurately classified patients with HCC in stage 0/A of Barcelona Clinic Liver Cancer (AUC = 0.9242) [38]. Studies have found that another scoring model, BALAD-2, consisting of these three markers combined with serum bilirubin and albumin, could stratify the HCC patients into distinct prognostic groups [39]. Furthermore, in 2008, the combined biomarker Japan Integrated Staging was proposed to provide better survival predictions for HCC patients [40]. These studies illustrated the potential of these three markers in the diagnosis and prognosis prediction of HCC. However, in the TCGA data we used, only the serum levels of AFP and DCP were provided. Therefore, we could not directly compare the predictive efficacy of our model with BALAD-2. We compared the AFP and DCP levels between the high- and low-risk groups predicted by our model. Results showed that these two proteins were not significantly differentially expressed between the training set's high- and low-risk groups (Wilcoxon–Mann–Whitney test: *p* = 0.486 and *p* = 0.771, respectively,). Elevated serum levels of AFP, AFP-L3, and DCP at baseline had been reported to be associated with a worse prognosis after resection of HCC [41]. We thus compared the corresponding mRNAs in the HCC patients, which showed that the mRNAs of the two markers were significantly up-regulated in the high-risk HCC samples compared to the normal controls (Wilcoxon–Mann–Whitney test: *p* < 0.05).

To further validate the predictive value of our prognostic PRlncRNA risk model, we compared its performance with three different prognostic models previously reported, which were also constructed based on PRlncRNAs using the same TCGA RNA sequencing data. Zhang et al. constructed a risk-scoring model of 5 PRlncRNAs, with 5-year AUCs of 0.688 for the training and 0.714 for the validation set, respectively [42]. Liu et al. built a prognostic risk-scoring model using 5 PRlncRNAs, with 5-year AUCs of 0.707 for the training set and 0.642 for the validation set, respectively [18]. The 5-year AUCs for the 9-PRlncRNA model built by Zhang et al. were 0.812 for the training set, and 0.722 for the validation set, respectively [43]. The predictive performance of our PRlncRNA prognostic model was higher than the three published models, with 5-year AUCs of 0.902 for the training set and 0.797 for the validation set, respectively.

The prognostic risk model in our study was constructed based on the REOs of PRlncRNAs. Although it is technically simpler to detect serum levels of protein markers such as the commonly used AFP, analysis at the RNA level may provide additional information for understanding cancer mechanistically. Most lncRNAs involved in the lncRNA–miRNA-PRG regulatory axes have been reported to be prognostically relevant in various cancers, including HCC. For example, LINC01554-mediated glucose metabolism reprogramming could suppress the tumorigenicity of HCC through the downregulation of PKM2 expression and inhibition of the Akt/mTOR signaling pathway [44]. NRSN2-AS1 could promote ovarian carcinogenesis through the miR-744-5p/PRKX axis [45]. Upregulation of lncRNA FOXD2-AS1 expression could promote the progression of HCC by causing epigenetic silencing of DKK1 and activating the Wnt/β-catenin signaling pathway [46]. Thus, these eleven lncRNA–miRNA-PRG regulatory axes could be helpful for further understanding the relationship between lncRNAs and PRGs and deserve further investigation.

## Conclusion

In summary, we propose an 11-PRlncRNA-pair personalized prognostic risk model for HCC, which let us see the robustness of the REO-based PRlncRNA prognostic biomarkers in the stratification of HCC patients at high and low risk. And the model is also helpful for understanding the molecular mechanisms between pyroptosis and HCC prognosis. High-risk patients may have excessive pyroptosis and thus be less sensitive to immune therapy.

## Supplementary Information

**Additional file 1. Table S1.** The 40 pyroptosis-associated genes identified from the MSigDB database.

**Additional file 2. Table S2.** Clinical characteristics of patients in training cohort and validation cohort.

## Data Availability

The original contributions presented in the study are included in the article or supplementary material, and further inquiries can be directed to the corresponding authors. The original data for the analysis in this study included RNA-seq data, miRNA-seq data and corresponding clinical data available at TCGA database (https://www.cancer.gov/about-nci/organization/ccg/research/structural-genomics/tcga), annotation information of all pathways available at the MSigDB database (https://www.gsea-msigdb.org/gsea/msigdb), information about miRNA-lncRNA interactions available at the miRNet database (https://www.mirnet.ca/), and information about PRG-miRNA interactions available at the TargetScan database (https://www.targetscan.org/vert_72/).

## Abbreviations

AFP: Alpha fetoprotein
AFP-L3: Lens culinaris agglutinin-reactive alpha-fetoprotein
AIC: Akaike information criterion
Akt: Protein kinase B
AUC: Area under the curve
BH: Benjamini–Hochberg
ceRNA: Competing endogenous RNA
Cox: Proportional hazards model
DCP: Des-γ-carboxy prothrombin
DKK1: Dickkopf WNT signaling pathway inhibitor 1
FDR: False discovery rate
FOXD2-AS1: FOXD2 adjacent opposite strand RNA 1
GSDMD: Gasdermin D
GSEA: Gene set enrichment analysis
GZMA: Granzyme A
GZMB: Granzyme B
GZMK: Granzyme K
HCC: Hepatocellular carcinoma
HOTTIP: HOXA distal transcript antisense RNA
ImmuCellAI: Immune Cell Abundance Identifier
LASSO: Least absolute shrinkage and selection operator
LINC01554: Long intergenic non-protein coding RNA 1554
lncRNA: Long noncoding RNA
MEG3: Maternally expressed 3
miR-744-5p: MicroRNA-744-5p
MSigDB: Molecular Signatures Database
mTOR: Mammalian target of rapamycin
NK: Natural killer
NLRP3: NLR family pyrin domain containing 3
NRSN2-AS1: NRSN2 antisense RNA 1
OS: Overall survival
PKM2: Pyruvate kinase M2
PRF1: Perforin 1
PRG: Pyroptosis-related gene
PRKX: Protein Kinase CAMP-Dependent X-Linked Catalytic Subunit
PRlncRNA: Pyroptosis-related lncRNA
REO: Relative expression ordering
RNA-Seq: Quantitative transcriptomics data
RPM: Reads per million
TCGA: The Cancer Genome Atlas
TPM: Transcript per million
Treg: Regulatory T cell
t-ROC: Time-dependent receiver operating characteristics
Wnt: Wingless/integrated
