# Supplementary material for: Screening prognostic markers for hepatocellular carcinoma based on pyroptosis-related lncRNA pairs
Source: BMC Bioinformatics. 2023 Apr 29;24:176. doi: 10.1186/s12859-023-05299-9 (PMC10148420; doi:10.1186/s12859-023-05299-9)
Supplement: Supplementary file 1 — Additional file 1. Table S1. The 40 pyroptosis-associated genes identified from the MSigDB database. [file 12859_2023_5299_MOESM1_ESM.pdf]

**Table S1 The 40 pyroptosis-associated genes identified from the MSigDB database**

| Pyroptosis-Related-Genes |
| --- |
| BAK1, BAX, CASP1, CASP3, CASP4, CASP5, CHMP2A, CHMP2B, CHMP3, CHMP4A, CHMP4B, CHMP4C, CHMP7, CYCS, ELANE, GSDME, HMGB1, IL1A, IL1B, IRF1, IRF2, TP53, TP63, AIM2, DHX9, GSDMA, GSDMB, GSDMC, GSDMD, GZMA, GZMB, NAIP, NLRP1, NLRP9, ZBP1 |
