# Supplementary material for: Screening prognostic markers for hepatocellular carcinoma based on pyroptosis-related lncRNA pairs
Source: BMC Bioinformatics. 2023 Apr 29;24:176. doi: 10.1186/s12859-023-05299-9 (PMC10148420; doi:10.1186/s12859-023-05299-9)
Supplement: Supplementary file 2 — Additional file 2. Table S2. Clinical characteristics of patients in training cohort and validation cohort. [file 12859_2023_5299_MOESM2_ESM.pdf]

Table 2 Clinical characteristics of patients in training cohort and validation cohort

	Training cohort		Validation cohort		P-value
	N = 240		N = 103		
	NO.	%	NO.	%	
**Age**					
<=60	119	49.6	46	44.7	0.472
>60	121	50.4	57	55.3	
**Gender**					
Female	77	32.1	33	32.0	1.000
Male	163	67.9	70	68.0	
**Stage**					
I	107	44.6	54	52.4	
II	58	24.2	19	18.4	
III	56	23.3	24	23.3	0.615
IV	2	0.8	1	1.0	
unknown	17	7.1	5	4.9	
**T**					
T1	111	46.3	57	55.3	
T2	64	26.7	20	19.4	
T3	52	21.7	23	22.3	0.426
T4	10	4.2	3	2.9	
unknown	3	1.3	0	0.0	
**N**					
N0	165	68.8	74	71.8	
N1	3	1.3	0	0.0	0.649
unknown	72	30.0	29	28.2	
**M**					
M0	166	69.2	79	76.7	0.287
M1	2	0.8	1	1.0	

unknown	72	30.0	23	22.3	
**Grade**					
G1	38	15.8	15	14.6	
G2	116	48.3	45	43.7	
G3	77	32.1	35	34.0	0.282
G4	5	2.1	7	6.8	
unknown	4	1.7	1	1.0	
